# Recovery of recombinant *Mycobacterium tuberculosis* antigens fused with cell wall-anchoring motif (LysM) from inclusion bodies using non-denaturing reagent (N-laurylsarcosine)

**DOI:** 10.1186/s12896-019-0522-x

**Published:** 2019-05-14

**Authors:** Anhar Danial Mustafa, Jeevanathan Kalyanasundram, Sarah Sabidi, Adelene Ai-Lian Song, Maha Abdullah, Raha Abdul Rahim, Khatijah Yusoff

**Affiliations:** 1 Department of Cell and Molecular Biology, Faculty of Biotechnology and Biomolecular Sciences, Universiti Putra Malaysia, 43400 Serdang, Selangor Malaysia; 2 Department of Microbiology, Faculty of Biotechnology and Biomolecular Sciences, Universiti Putra Malaysia, 43400 Serdang, Selangor Malaysia; 3 Department of Pathology, Faculty of Medicine and Health Sciences, Universiti Putra Malaysia, 43400 Serdang, Selangor Malaysia; 4 Institute of Bioscience, Universiti Putra Malaysia, 43400 Serdang, Selangor Malaysia; 5 Malaysia Genome Institute, 43000 Kajang, Selangor Malaysia

**Keywords:** *Mycobacterium tuberculosis* antigen, Lysine motif, Overexpression, Inclusion body, TB subunit vaccine, N-lauroylsarcosine

## Abstract

**Background:**

The current limitations of conventional BCG vaccines highlights the importance in developing novel and effective vaccines against tuberculosis (TB). The utilization of probiotics such as *Lactobacillus plantarum* for the delivery of TB antigens through in-trans surface display provides an effective and safe vaccine approach against TB. Such non-recombinant probiotic surface display strategy involves the fusion of candidate proteins with cell wall binding domain such as LysM, which enables the fusion protein to anchor the *L. plantarum* cell wall externally, without the need for vector genetic modification. This approach requires sufficient production of these recombinant fusion proteins in cell factory such as *Escherichia coli* which has been shown to be effective in heterologous protein production for decades. However, overexpression in *E. coli* expression system resulted in limited amount of soluble heterologous TB-LysM fusion protein, since most of it are accumulated as insoluble aggregates in inclusion bodies (IBs). Conventional methods of denaturation and renaturation for solubilizing IBs are costly, time-consuming and tedious. Thus, in this study, an alternative method for TB antigen-LysM protein solubilization from IBs based on the use of non-denaturating reagent N-lauroylsarcosine (NLS) was investigated.

**Results:**

Expression of TB antigen-LysM fusion genes was conducted in *Escherichia coli,* but this resulted in IBs deposition in contrast to the expression of TB antigens only. This suggested that LysM fusion significantly altered solubility of the TB antigens produced in *E. coli*. The non-denaturing NLS technique was used and optimized to successfully solubilize and purify ~ 55% of the recombinant cell wall-anchoring TB antigen from the IBs. Functionality of the recovered protein was analyzed via immunofluorescence microscopy and whole cell ELISA which showed successful and stable cell wall binding to *L. plantarum* (up to 5 days).

**Conclusion:**

The presented NLS purification strategy enables an efficient and rapid method for obtaining higher yields of soluble cell wall-anchoring *Mycobacterium tuberculosis* antigens-LysM fusion proteins from IBs in *E. coli*.

**Electronic supplementary material:**

The online version of this article (10.1186/s12896-019-0522-x) contains supplementary material, which is available to authorized users.

## Background

Tuberculosis (TB) is one of the leading causes of morbidity and mortality in humans, and it represents a major public health problem in many developing and underdeveloped countries [1]. Almost one third of the world population is latently infected with TB with around 1.6 million deaths recorded in 2017 [2]. Thus, the prevention of TB is crucial particularly when the current standard vaccine, Bacille Calmette-Guérin (BCG) vaccine, has shown to be suboptimal and less effective (0–80%) in tropical and sub-tropical regions [3]. This underlines the urgency in developing second generation vaccines which could function as a competent prophylactic vaccine and/or a booster vaccine that improves immunity in BCG-vaccinated individuals.

A mucosal antigen delivery strategy using probiotic lactic acid bacteria such as *Lactobacillus plantarum* as a carrier has the potential to be developed as an effective TB vaccine. It was reported that the presence of *L. plantarum* with *M. tuberculosis* antigens can act as an adjuvant and help improve the immune response to a favorable Th1 response, prerequisite for effective humoral and cell-mediated immunity [4]. Other advantages of using *L. plantarum* as a mucosal vaccine delivery vehicle has been described previously [5–7]. These antigen carriers are able to persist and colonize certain regions in the mucosa. The surface display of antigenic protein can be expressed by the bacteria intracellularly and secreted for outer cell wall attachment [8, 9]. Alternatively, antigenic protein can also be surface displayed in-trans, by first producing the protein fused with binding domain in a separate expression system such as in *Escherichia coli*. The target fusion protein will then be purified and subsequently introduced externally to *L. plantarum* for cell surface anchoring [7, 10, 11]. The latter method produces a non-genetically modified organism (GMO) bacterial vaccine, which provides a safer vaccine option particularly with impending post-antibiotic era. However, an appropriate anchor protein is required for both secretion and in-trans surface display approach. For instance, the LPXTG anchor motif that binds covalently to peptidoglycan can only be used in the first binding approach [12], while the lysine motif protein domain (LysM) is more suitable for the second binding approach as it binds non-covalently to the peptidoglycan layer [7, 13].

In recent years, extensive progress on TB subunit vaccine research has produced several promising vaccines which are currently being tested at different stages of clinical trials [14]. These subunit vaccines consist of effective immunodominant TB proteins, including Ag85B, Rv2031 and Rv0475. The Ag85B is a mycolyltransferase that is able to induce both humoral and cell-mediated immune responses in *M. tuberculosis*-infected subjects [15]. The Rv2031 or HspX, on the other hand, is a stress protein, induced by anoxia which has a proposed role in maintaining long-term viability during latent and asymptomatic infections [16, 17]. The Rv0475 or HbhA is a protein required for extrapulmonary dissemination [16]. These proteins are considered immunodominant proteins as they have been demonstrated to induce strong immune responses in different animal models [18] as well as T cell induction in human infected with *M. tuberculosis* [17]. The combination of these immunodominant antigens within multiple epitope fusion proteins, similar to the current TB subunit trial vaccines, have been shown to extensively improve the immunogenicity and protective efficacy of subunit vaccine. This is due to the higher potential of immune-reactivity of multiple immunogenic proteins compared to a single immunogenic protein [18].

*E. coli* is the most common host cell for efficient and safe expression of native or recombinant antigens from *M. tuberculosis* [18]. Although protein production can be readily optimized in *E.coli*, overexpression generally leads to a significant amount of the protein being misfolded or/and aggregated [19]. Hitherto, most recombinant TB vaccine candidates have been purified at a significant lower amounts since most of them were detected to be accumulated in inclusion bodies (IBs) [15, 18]. These protein are usually recovered from the IBs by initial denaturation with urea or guanidine hydrochloride followed by protein renaturation steps [20]. This method is expensive, tedious and often resulted in low recovery yields of 15–25% [19]. Another alternative approach is to use a mild detergent, such as N-lauroylsarcosine (NLS), which has been shown previously, to effectively recover soluble properly folded proteins from IBs formed in *E. coli* under non-denaturing condition [20–22].

In this study, the cell wall-anchoring recombinant protein ARL was constructed and expressed in *E. coli*. The ARL protein consists of *M. tuberculosis* Ag85B_101–115,126-140,261–275_, Rv0475_34–59_ and Rv2031_41–70, 95–108_ epitopes (AR) fused with the LysM anchoring domain of AcmA, a N-acetylglucosaminidase of *Lactococcus lactis* MG1363 [23]. However, it was observed that the fusion protein ARL was accumulated in IBs. Thus, the aim of this study, was to recover a soluble form of the ARL protein from the IBs using the NLS treatment. Once the target protein was recovered, the binding capability of ARL was tested via in vitro binding studies onto *L. plantarum* Pa21 cell surface. The retaining of the ARL-LysM cell wall binding ability, helps to determine the suitability of such pre-treatment assay for recovering soluble TB fusion protein from the IBs in the future. The NLS pre-solubilization step for IBs of other *M. tuberculosis* antigenic proteins fused with cell wall-anchoring motif such as LysM may be advantageous since this approach is rapid, cheaper and more effective than the conventional denaturing/renaturing method.

## Results

### Development and overexpression of pRSF:ARL

**Fig. 1:**
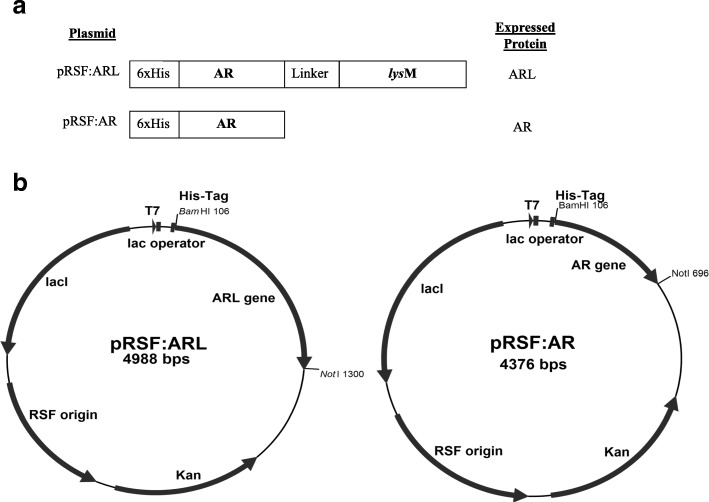
**a** Schematic illustration of the fusion proteins encoded by pRSF:ARL expressing the LysM binding motif from *L. lactis* AcmA, and pRSF:AR missing the *lys*M sequence. **b** Map of the recombinant plasmids pRSF:ARL and pRSF:AR. Both ARL and AR genes were inserted between *Bam*HI and *Not*I sites of pRSF:Duet-1 to construct pRSF:ARL and pRSF:AR expression plasmid, respectively

**Fig. 2:**
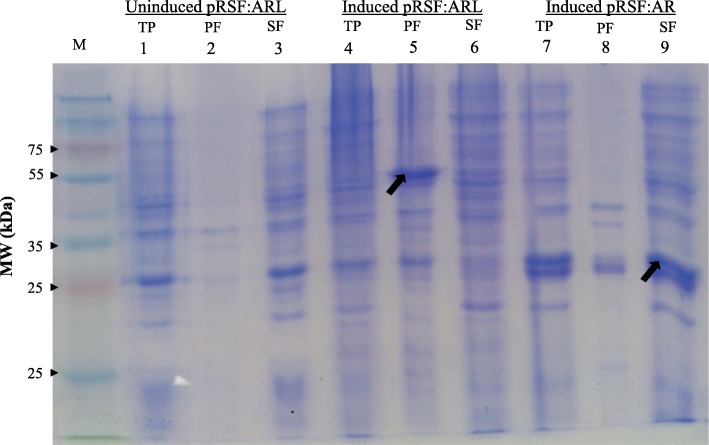
Expression analysis of the ARL protein in *E. coli*. Positive transformant cells harbouring pRSF:ARL were induced, lysed and analyzed for protein expression by sodium dodecyl sulfate polyacrylamide (SDS-PAGE). A control batch of bacterial cells harbouring pRSF:AR was also induced and analyzed to compare AR (~ 24 kDa) solubility to that of ARL (~ 45 kDa). Lanes 1–3, 4–6 and 7–9 correspond to the protein profile of uninduced cells harbouring pRSF:ARL, induced cells harbouring pRSF:ARL, and induced cells harbouring pRSF:AR, respectively. Lanes labeled with M, TP (lanes 1, 4 and 7), PF (lanes 2, 5 and 8) and SF (lanes 3, 6 and 9) refer to protein ladder marker (Fermentas, Canada), total protein, precipitant fraction and supernatant fraction, respectively

The construction of pRSF:ARL and pRSF:AR (Fig. 1) was successfully achieved as verified via *Bam*HI/*Not*I restriction enzyme analysis (Additional file 1: Figure S1) and sequence analyses (Additional file 2: Figure S2). Overexpression of ARL and AR, respectively, was obtained using pRSFDuet-1 vector in *E. coli* Rosetta (DE3) pLySs strain using IPTG as inducer (Fig. 2). Without induction, ARL was not observed in either insoluble (precipitant) or soluble (supernatant) fractions as indicated in Fig. 2 of lanes 2 and 3, respectively. Following induction with IPTG, an intense band was observed in the precipitant fraction (Fig. 2, lane 5) indicating that the overexpressed ARL was mostly deposited into IBs as opposed to the soluble or supernatant fraction (Fig. 2, lane 6). Interestingly, the total protein fraction (Fig. 2, lane 4) did not show visible ARL target protein band. This may be due to the concentration of ARL which was too low and therefore masked by host endogenous proteins compared to the precipitant fraction, a concentrated fraction containing mostly the ARL IBs. This was supported by the Western blot results where a clear band corresponding to ARL protein was observed in the total fraction sample. (Additional file 3: Figure S3). Densitometry analysis revealed that the overexpressed ARL protein in the insoluble fraction with the expected molecular weight of ~ 45 kDa (Fig. 2, lane 5), was ~ 47% pure. Unlike the ARL protein, the AR protein of 24 kDa was more prominently present in the soluble cell fraction (Fig. 2, lane 9) than in the insoluble cell fraction (Fig. 2, lane 8), suggesting that the fusion with LysM protein had affected the solubility of the expressed ARL.

### Extraction of ARL from IBs using NLS

**Fig. 3:**
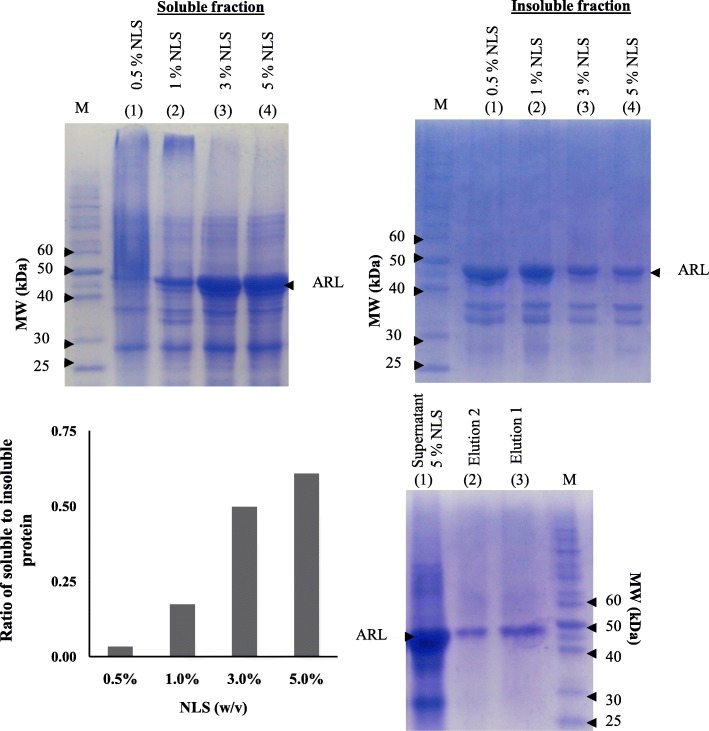
Solubilization and purification of ARL inclusion bodies (IBs) using varying concentrations of 0.5 1, 3 and 5% (w/v) NLS **a** Soluble fractions of ARL after NLS treatment. **b** Insoluble fractions of ARL after NLS treatment. **c** Ratio of soluble protein to insoluble protein of ARL obtained from IBs solubilized with varying NLS concentration. **d** 5% NLS-treated ARL which was diluted to 0.1% NLS initially before the purification via the Ni^2+^-NTA affinity chromatography

Effective NLS concentration is important to solubilize and extract maximum amount of target proteins from IBs. Thus, to achieve the optimal NLS concentration, treatment was carried out using ARL protein with varying NLS concentration of 0.5, 1, 3, and 5% (w/v) for 24 h at 20 °C as depicted in Fig. 3a. The ARL IBs solubility improved with the increase of NLS concentration up to 5% (w/v) NLS (Fig. 3c). The results also implied that a NLS concentration above 5% would have little impact in improving the solubilization of ARL IBs. This is because the ratio of soluble/insoluble protein solubilization of ARL IBs seems to reach a plateau value when higher NLS concentration was used (Fig. 3c) as shown by the ratio between NLS 3% (0.498) and 5% (0.609). This also indicated that there was still a fraction of partially or totally misfolded proteins present inside the IBs, which were unaffected by the NLS solubilization treatment. Based on the densitometry analysis, the extractability of ARL IBs into the soluble form was most favorable with 5% (w/v) NLS. At this concentration, ARL IBs were effectively solubilized (Table 1). NLS-solubilized ARL was then diluted to a final concentration of 0.1% NLS before being applied onto the Ni^2+^-NTA matrix column as to avoid inhibition of binding to the Ni^2+^-NTA- matrix by NLS [20]. Approximately 55.4% of IBs containing ARL protein was able to be solubilized and bound to Ni^2+^-NTA matrix before being eluted with elution buffer containing 500 mM imidazole in two fractions (Elution 1 and Elution 2) which was combined afterwards (Fig. 3d). This one-step purification resulted in highly pure ARL protein with more than 98% purity. During the solubilization and purification process, the extractability of ARL IBs yielded a purified protein of 0.63 mg per 0.1 g cell mass. Based on the SDS-PAGE analysis, the molecular weight of the purified ARL was estimated to be approximately 45 kDa (Fig. 3d), similar to that of the expected ARL protein size. Protein recovery at every step of 5% (w/v) NLS solubilization and purification is shown in Table 1.

**Table 1:** ARL protein recoveries (per 0.1 g wet cell mass) from inclusion bodies (IBs) by 5% (w/v) N-lauroylsarcosine (NLS) and its purification by Ni^2+^-NTA affinity chromatography

Stages of solubilization/purification	Total protein (mg)/0.1 g cell wet mass	Target protein (mg)/0.1 g cell wet mass	Purity (%)
Cell lysate	3.21	1.53	47
Insoluble fraction/IBs	1.9	1.14	60
Soluble fraction after 5% NLS treatment	1.12	0.69	61.8
Purified ARL after Ni^2+^-NTA affinity chromatography	0.64	0.63	98
Extractability (%) from IBs	33.68%	55.42%	–

### Binding capability of ARL onto *L. plantarum* cell wall

**Fig. 4:**
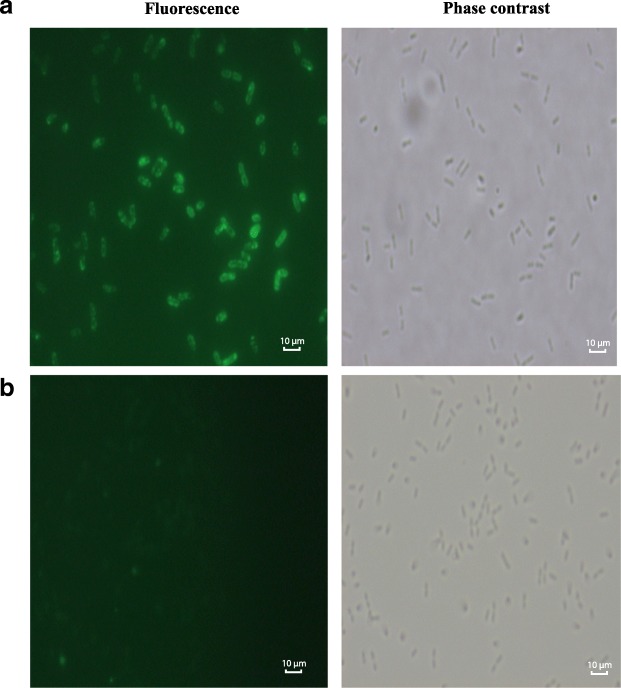
Binding of ARL onto *L. plantarum* Pa21 cells. Fluorescence and phase contrast microscopy of *L. plantarum* cells incubated with **a** ARL protein and **b** PBS. The positive FITC signalling (designated by the green fluorescence) on *L. plantarum* cells incubated with ARL protein indicates successful ARL attachment to *L. plantarum* cell wall. The cells were observed under 100x objective in all frames

In order to determine the binding capability of the solubilized ARL proteins, attachment of the purified ARL proteins onto *L. plantarum* cell wall was performed and subjected to immunofluorescence staining for qualitative evaluation and confirmation. As shown in Fig. 4a, ARL protein was successfully bound to the cell wall of *L. plantarum.* Bright fluorescence highlighting on the rod-shaped *Lactobacilli* cells was observed using phase contrast imaging indicating a successful attachment of ARL on the cell wall surface of the bacteria. Meanwhile, the negative control comprising bacterial cells suspended in PBS (Fig. 4b) showed no fluorescence signal.

**Fig. 5:**
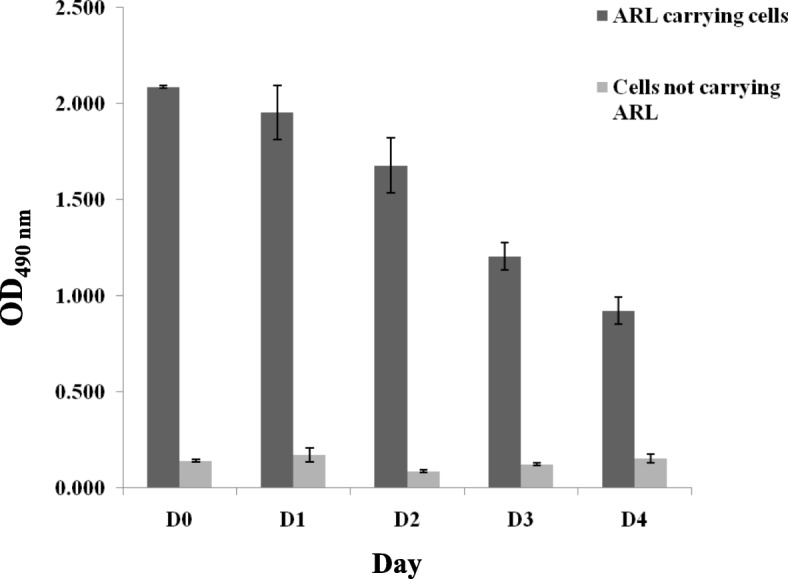
Stability analysis of the ARL protein anchored onto cell surface of *L. plantarum*. The cells were incubated with ARL protein and were subjected to ELISA using mouse anti-His monoclonal IgGs (Novagen, USA) as primary antibody and goat anti-mouse IgG-HRP (Calbiochem, USA) as secondary antibody for the duration of 5 days (Day 0 to Day 4). Negative control was *L. plantarum* incubated with PBS

To semi-quantitatively investigate and determine whether the ARL purified proteins were able to be stably anchored and displayed onto the *L. plantarum* cell surface, whole cell ELISA was performed over 5 days period (Fig. 5). The values of the absorbance (OD_490_ nm) correlated to the frequency by which ARL had attached onto the cell walls of *L. plantarum*, thus projecting the ARL binding stability each day from Day 0 to 4. The ARL attached to *L. plantarum* showed a consistent pattern of binding stability that involved a slight reduction in absorbance reading at Day 2 followed by a gradual decrease of absorbance until Day 4 (Fig. 5).

## Discussion

Inclusion bodies (IBs) in *E. coli* are dense and porous particles containing almost exclusively overexpressed and aggregated proteins [19]. The prevalence of protein aggregation in *E. coli* can be due to overexpression and/or misfolded heterologous proteins. The overexpression of heterologous of protein has been posited to disrupt intracellular proteostasis which triggers formation of IBs a part of *E. coli* stress response, in order to retain protein equilibrium [24]. On the other hand, the irregularities in protein folding, especially misfolded proteins, tend to reveal higher hydrophobic regions which lead to rapid protein interaction thus promoting aggregation [25]. The common strategy of IBs solubilization uses denaturants such as urea and guanidine-hydrochloride with additional renaturation steps. This conventional method requires the linearization of the IBs protein via the removal of the disulfide linkages before allowing gradual refolding of the protein under optimized renaturing agent concentration with stabilizer and additive reagents [19]. However, hydrophobic interactions and incorrect disulfide bond formations severely damages protein renaturation [20]. Alternatively, the use of NLS, an ionic and non-denaturant detergent, has been shown previously to effectively solubilized IBs composed of various protein types [20–22]. Nonetheless, solubilization of IBs using NLS appears to be only efficient against IBs harbouring partially folded protein aggregates [21]. Based on the proposed mechanism for IBs solubilization by NLS, the latter compound acts by penetrating the IBs pores and inhibiting aggregation of proteins via encapsulation of the proteins [22] and masking of their hydrophobic patches [20], thus canceling the interaction between partially folded proteins. On the other hand, IBs composed of primarily misfolded proteins are inherently insoluble and can only be solubilized with the conventional method.

The overexpression of ARL protein was achieved using the expression vector pRSFDuet-1 in *E. coli* which is based on an IPTG-inducible expression system. However, most of the expressed ARL protein were detected to be accumulated in IBs. This is in contrast to the AR protein, which was produced as a soluble protein under same expression system (see Fig. 2), suggesting that the fusion of LysM with AR protein may have affected ARL solubility. This formation of IBs can be a significant obstacle for the development of surface displayed, cell wall-anchored subunit TB vaccines especially in obtaining optimal quantity of proteins with correct conformational structure. Previous studies utilizing a similar approach of expressing various types of antigen-LysM fusions in *E. coli* expression host [7, 26–28] have yet to encounter this issue. There are various possible reasons for the fusion of LysM to AR which causes the resulting fusion protein to be deposited into IBs. It is possible that an increase in protein size as a result of the AR-LysM fusion may affect the efficiency of proper refolding in *E. coli* [29]. Moreover, the combination between AR and LysM as fusion protein may not be compatible and may cause protein-protein interaction effect that leads to improper and partial protein folding conformation which promotes protein aggregation and IBs formation [29].

The ARL IBs were solubilized using various NLS concentrations ranging from 0.5–5% (w/v). Although a higher NLS concentration of 10% was reported to be able to solubilize > 95% of IBs, subsequent dilution to < 0.3% final concentration for Ni^2+^–NTA column purification and downstream processes destabilized the protein and promotes aggregation [22]. Therefore, a lower range of NLS concentration up to 5% was tested instead. Among the NLS concentrations tested, 5% NLS (w/v) was determined to be the most favorable NLS concentration for ARL IBs solubilization. In order to capture all of the solubilized protein on the affinity column, the NLS concentration needs to be less than 0.3% [21] since higher NLS concentration prevents effective binding of protein onto the column due to the masking of the His tag by the detergent [20]. However, the extreme dilution of the NLS from 5% (w/v) to 0.3% (w/v) post IBs solubilization in order to promote Ni^2+^–NTA column protein capturing, also has its limitation. The swift reduction of NLS concentration may impair its ability to prevent protein re-aggregation and precipitation. This limitation poses a major hurdle in upscaling fusion protein production in order to obtain suitable amount of protein for downstream process. [22]. Hence, the pretreatment NLS concentration in this study was diluted to 0.1% (w/v) to improve capture of NLS-treated ARL to Ni^2+^–NTA column without the need for diluting the solubilized protein sample. Based on this single-step purification method, the protein recovery yield was approximately 55% at more than 98% purity. A final yield of 0.63 mg of purified protein could be obtained per 0.1 g wet cell mass.

The functionality and stability of ARL to anchor to the cell wall of *L. plantarum* was then analysed via immunofluorescence microscopy and whole cell ELISA for qualitative and semi-quantitative analysis, respectively. Both methods showed that the binding of ARL to the *L. plantarum* cell wall was successfully achieved. Attachment of ARL to the cell wall of *L. plantarum* was due to the triple LysM domain that binds non-covalently to peptidoglycan on the cell wall surface of *L. plantarum* [13]. Interestingly, the LysM motif can recognize and attach to a broad range of bacterial cell walls particularly of Gram-positive bacteria, such as *Clostridium*, *Listeria*, and *Bacillus* [11]. This feature is advantageous in making the ARL protein flexible for further optimization using a variety of bacteria as vaccine delivery vehicles. The ARL binding stability showed similar pattern as in other studies [7, 26] where consistent binding within a period of several days was observed. However, as shown in Fig. 5, gradual decrease of the absorbance values from the initial binding at Day 0 over the period of 5 days indicated that the binding stability of ARL may be reduced after a longer period of time. This reduction of ARL binding may be a consequence of proteolysis by *Lactobacilli* housekeeping proteases such as HtrA [30], which cleaves surface adhesion protein including that of the attached ARL protein on the *Lactobacilli* cell wall. Moreover, the occurrence of cell division may also result in the detachment of bound ARL to *L. plantarum* cell wall, thereby disrupting ARL binding stability. Ultimately, the successful cell wall binding of ARL acts as preliminary indicator that the solubilization of ARL IBs using NLS has resulted in functional protein conformation. Although this approach only focuses on the binding capability of ARL, it is postulated that the correlation between correctly folded protein and the functional LysM domain after NLS solubilization indicates strongly that the antigenic part (AR) of the protein may have retained its native and proper protein conformation which is important in triggering prophylactic immune response against TB. Hence, the immunogenicity of ARL attached to *L. plantarum* needs still to be investigated in future particularly via the in vivo studies.

## Conclusions

In summary, an efficient and rapid method for obtaining soluble cell wall-anchoring *M. tuberculosis* ARL from IBs using NLS, is described. This purification strategy can potentially be used for the purification of other cell wall-anchoring fusion proteins that are expressed as IBs. It is recommended that this strategy is applied first as the reported method is a rapid and cost-effective option than that of the conventional method of IBs extraction via denaturation and renaturation procedures. If the NLS treatment still does not provide any significant recovery of soluble protein, the IBs are most likely composed of totally misfolded proteins, and thus requires the conventional method instead.

## Methods

### Microorganisms and plasmids

*Lactococcus lactis* MG1363 [23] was inoculated at 30 °C in M17 medium (Sigma, USA) before its genomic DNA was extracted and used for the amplification of the *lys*M binding motif of the *acm*A gene (accession no. U17696.1). *Lactobacillus plantarum* Pa21 cells [31] were used as the carrier vehicle to display *M. tuberculosis* antigens. *Escherichia coli* Rosetta (DE3) pLysS (Novagen, USA) was used as the expression host cell in combination with pRSF:DuetT-1 (Invitrogen, USA) as the expression vector for the recombinant genes. The *M. tuberculosis* antigens of Ag85B (accession no. Q847N4), Rv0475 (accession no. CCP43209) and Rv2031 (accession no. AJF03385.1) were synthesized based on their epitope-encoding sequences of Ag85B_101–115,126-140,261–275,_ Rv0475_34–59_ and Rv2031_41–70,95–108_ proteins and assembled into one fusion gene designated as AR before being cloned into pJET1.2/blunt cloning vector (Integrated DNA Technologies, Singapore). The epitope-encoding sequences were selected based on their bioinformatics analysis that showed strong potential for inducing effective and protective immune response against TB. The relevant features of bacterial strains and plasmids used in this study are described in Table 2.

**Table 2:** Bacterial strains and plasmids used in this study

Strain or plasmid	Relevant Characteristics	Source or reference
Strains		
*Lactococcus lactis* MG1363	*L. lactis* subsp. *cremoris*; Lac^−^, plasmid-free derivative of NCDO712	[23]
*Lactobacillus plantarum* Pa21	Malaysia local plant, *Pandanus amaryllifolius*isolate, plasmid-free strain	[31]
*E. coli* Rosetta (DE3)pLysS	Cam^r^, Expression host for all *E. coli* vectors, *E. coli* BL21 derivative	Novagen
Plasmids		
pRSFDuet-1	3.8 kb, Kan^r^, *E. coli* expression vector	Invitrogen
pJET1.2/blunt	2.9 kb, Amp^r^, *E. coli* cloning vector,	Thermo Scientific
pJET:AR	3.5 kb, Amp^r^, pJET1.2 derivative carrying AR gene	IDT
pRSF:ARL	4.98 kb, Kan^r^, pRSFDuet-1 derivative carrying ARL fusion gene	This study
pRSF:AR	4.4 kb, Kan^r^, pRSFDuet-1 derivative carrying AR fusion gene	This study

### DNA techniques and transformation

The methods for molecular cloning were performed according to Sambrook et al. [32]. Genomic DNA of *L. lactis* was isolated according to Leenhouts et al. [33]. Plasmid DNA was obtained using Promega Mini-Prep Plasmid Extraction kit (Promega, USA) as specified by the supplier. Restriction enzymes, T4 DNA ligase, and deoxynucleotides were obtained from Roche Diagnostics (Germany) and were used according to the supplier’s instructions. Polymerase chain reactions (PCR) were carried out via T100 Thermal Cycler (BioRad, USA) using Pfu DNA polymerase according to the instructions of the manufacturer (Fisher Scientific, USA). PCR products were purified using the Wizard® SV Gel and PCR Clean-Up System (Promega, USA) and was performed according to manufacturer’s protocol. *E. coli* Rosetta strain was transformed with the recombinant vector by the heat-shock method.

### Construction and expression of plasmid pRSF:ARL

In order to construct the pRSF:ARL expression plasmid, AR and *lys*M DNA sequences were individually amplified using pre-designed primers (Table 3) from pJET:AR and from genomic DNA of *L. lactis* MG1363*,* respectively. The PCR reactions were carried based on these parameters; denaturation step at 95 °C for 5 min and amplification in 25 cycles of 1 min at 95 °C, 30 s at optimum annealing temperature, which was determined by gradient temperature ranging from 45 to 60 °C, and 1 min at 72 °C followed by one cycle at 72 °C for 10 min. Gel purified PCR products of AR and *lys*M were digested with *Pst*I, purified and ligated together at a 1:1 ratio using T4 ligase (Roche, Germany) to produce ligated product of the ARL gene. Subsequently, the ARL fusion gene was PCR amplified and cloned into pRSFDuet-1 plasmid. Both insert and plasmid were double digested with *Bam*HI/*Not*I before ligation at 14 °C overnight at 1:4 plasmid/insert ratio. The newly constructed plasmid pRSF:ARL was then introduced into *E. coli* Rosetta (DE3) pLySs. After transformation, verification of the recombinant plasmid was performed by restriction enzyme analysis and PCR, and the inserted ARL gene was validated for its sequences via sequencing analysis approach (First Base, Malaysia). Plasmid pRSF:AR without the *lysM* sequence was constructed similarly to pRSF:ARL. The resulting plasmids pRSF:ARL and pRSF:AR are illustrated in Fig. 1.

**Table 3:** Primers used in this study

Fragment	Primer sequence (5′ to 3′)^a^	Expected product size (base pair)
ARL	Forward **GGATCC**GAATTCGCTGACCAGCGAGCTGCCGC	1244
	Reverse **GCGGCCGC**TTATTTTATTCGTAGATACTGAC	
*lys*M	Forward **CTGCAG**CCATGGCGGCTGGAAGACGAGATGAAA	608
	Reverse **GCGGCCGC**TTATTTTATTCGTAGATACTGAC	
AR	Forward **GGATCC**GAATTCGCTGACCAGCGAGCTGCCGC	613
	Reverse **GCGGCCGC**TGGCTTCCCTTCCGAAACCGC	
	Reverse **CTGCAG**GCTGGCTTCCCTTCCGAAACCGC	
pRSF:Duet-1	Forward GGATCTCGACGCTCTCCCT	200
	Reverse TTGTACACGGCCGCATAATC	

^a^Restriction enzyme (RE) sites are shown as bold and underlined is either *Bam*HI (GGATCC), *Pst*I (CTGCAG) or *Not*I (GCGGCCGC)

Two hundred-mL cultures of Terrific Broth (TB) (EMD BioSciences, San Diego, CA) medium with kanamycin (100 μg/ mL) were used to over-express the *E. coli* transformants containing pRSF:ARL and pRSF:AR, respectively. The cultures were grown at 37 °C until OD_600_ 0.5–0.7 before they were induced by adding IPTG (1 mM final concentration) for 6 h at 25 °C with 150 rpm shaking. The cells were then harvested by centrifugation for 10 min at 5000 g at 4 °C. The cell pellets (~ 0.1 g) were re-suspended in 10 mL PBS (pH 7.4) with sonication for 7 min (1 s on/2 s off) at 40% pulse mode power via the Omni Ruptor 4000 (Omni International, USA). The soluble and insoluble fractions were separated by centrifugation at 4 °C for 20 min at 5000 g, and analyzed in 12% SDS-PAGE. Target protein was estimated by densitometric analysis using ImageJ software [34].

### Solubilization and purification of ARL

The solubilization and purification of ARL was performed according to Mustafa et al. [35]. NLS concentrations of 0.5, 1, 3% or 5% (w/v) in 10 mL solubilizing buffer (40 mMTris–HCl, pH 8) were used to treat the re-suspended insoluble fraction (containing IBs) under constant agitation at 180 rpm for 24 h at 20 °C. Centrifugation of the suspension was carried out at 4400 g for 20 min at 4 °C and the supernatant was filter sterilized with 0.45 μm membrane filter. In order to achieve a final NLS concentration of 0.1% (w/v), binding buffer (20 mM imidazole, 40 mM Tris-HCl) was added to the supernatant accordingly with its pH adjusted to pH 7.4. Subsequently, the supernatant was loaded onto a Ni^2+^–NTA column (GE Healthcare, USA) followed by a washing step, three times with binding buffer (20 mM imidazole, 40 mM Tris-HCl) before being eluted with elution buffer (500 mM imidazole, 40 mM Tris-HCl). Analysis of the eluted protein and its concentration was determined based on SDS-PAGE analysis and Bradford assay, respectively.

### Binding of ARL onto *L. plantarum* cell wall surface

A 5 mL MRS broth (Difco, Detroit, MI) was inoculated with a single colony of *L. plantarum* at 37 °C in an overnight incubation. The 0.1 mL overnight culture was added into a new 5 mL MRS for inoculation at 37 °C. The cells culture was grown until it reached optical density of OD_600_ 0.5–0.7. The cells were then pelleted at 4000 g for 5 min and the cell pellet was re-suspended in 1 mL of MRS broth. The 1 mL of bacterial suspension was then mixed with 250 μg of purified ARL and incubated at 37 °C for 2 h. The cells were pelleted at 2000 g for 5 min and then washed with PBS for 3 times. Finally, the pellet was re-suspended in 1 mL of PBS and stored at 4 °C until further usage. The sample was further analyzed via immunofluorescent staining and whole cell ELISA assays as described below. *Lactobacilli* cells mixed with 200 μL of PBS were used as negative control.

### Immunofluorescence microscopy and ELISA

The qualitative confirmation of ARL binding to the *L. plantarum* cell wall was conducted at Day 0 via immunofluorescence visualization method using mouse anti-his IgG monoclonal antibody (Novagen, USA) as the primary antibody and goat anti-mouse IgG conjugated Fluorescein Conjugated Antibody (FITC)- (Calbiochem, USA) as the secondary antibody. Following the attachment procedure, the *Lactobacilli* cells were harvested and re-suspended in 300 μL of PBS. About 20–30 μl of 1 × 10^8^ cfu/mL cells was dropped on slides coated with poly-L-lysine; air dried and then washed with PBS. The attached cells were fixed with 4% (w/v) paraformaldehyde for 20 min at RT, followed with a washing step (3x), each for 5 min. In the next step, the cells were incubated with 3% (w/v) BSA in PBS for 30 min at RT, to block non-specific binding sites. After washing with PBS, the cells were incubated with primary antibody of anti-his monoclonal antibody (0.2 μg/μL) (Novagen, USA) diluted at ratio 1:200 in PBS with 1% BSA followed by incubation at RT for 1 h. The cells were washed and then incubated with secondary antibody goat anti-mouse IgG-FITC (1 μg/μL) (Calbiochem, USA) diluted at 1:200 in PBS with 1% BSA, at RT for 1 h. After the washing step, the slide was dried and analyzed by a fluorescence microscope (Nikon E200, Japan).

Whole cell ELISA was used to semi-quantitatively verify the binding stability and frequency of ARL protein attached onto the cell wall of *L. plantarum* for 5 days. At Day 0, 200 μL from the stored sample of *Lactobacilli* cells formerly incubated with the ARL proteins were harvested at 2000 g for 5 min. After that the cells were fixed in a 1 mL Eppendorf tube (Sigma, USA) with 4% (w/v) paraformaldehyde for 20 min at RT, followed by 3 times washing with PBS at 2000 g for 5 min. The cells were then incubated with blocking solution [3% (w/v) BSA in PBS] for 30 min at RT. After washing, the cells were incubated with mouse anti-his monoclonal antibody (0.2 μg/μL) (Novagen, USA) as primary antibody at a ratio of 1:200 in PBS with 1% BSA, followed by 1 h incubation at RT. The cells were then pelleted washed and incubated with horseradish peroxidase (HRP)-conjugated goat anti-mouse IgG antibody (1 μg/μL) (Novagen, USA) as secondary antibody at a ratio of 1:200 in PBS with 1% BSA for 1 h at RT. After washing, the cells were pelleted and finally re-suspended in 200 μL of PBS. Cells were then normalized to OD_600_ of 3 where after an appropriate volume of the bacterial suspension was added onto the 96 well ELISA plate (Sigma, USA). Subsequently 50 μL of substrate (BM Blue, Roche, Germany) was mixed in the wells of the ELISA plate and then incubated at RT for 20 min followed with the addition of 50 μL of stop solution (1 M H_2_SO_4_). The absorbance was measured at 490 nm using an ELISA reader (Tecan Infinite F50, Switzerland). On 5 subsequent days, an aliquot of the cells was taken and cells were subjected to the same treatment after fixation with 4% (w/v) paraformaldehyde as mentioned earlier. For both IF and whole cell ELISA, *Lactobacilli* cells incubated with PBS was used as negative control.

### Statistical analysis

All tests were performed using the student T-test function of the StatView program, version 5.0 (SAS Institute Inc.; Cary, NC). All results were valued as means± standard error.

## Additional files

Additional file 1: **Figure S1.** Confirmation of ARL and AR insert in pRSF:Duet plasmid by double restriction enzyme digests. (DOCX 692 kb)

Additional file 2: **Figure S2.** Sequencing results for ARL and AR in pRSF:Duet plasmid. (DOCX 48 kb)

Additional file 3: **Figure S3.** Western blot analysis of ARL from the total protein fraction sample. (DOCX 60 kb)

## Abbreviations

AR: Ag85B-Rv0475-Rv203
ARL: Ag85B-Rv0475-Rv203-LysM
BCG: Bacillus Calmette–Guérin
BSA: Bovine serum albumin
ELISA: Enzyme-linked immunosorbent assay
FITC: Fluorescein isothiocyanate
GMO: Genetically modified organism
HRP: Horseradish peroxidase; SDS-PAGE, sodium dodecyl sulfate polyacrylamide gel electrophoresis
IBs: Inclusion bodies
IgG: Immunoglobulin G
LAB: Lactic acid bacteria
LysM: Lysin motif
NLS: N-laurylsarcosine
PBS: Phosphate buffered saline
RT: Room temperature
Th1: T-helper 1
