# Supplementary material for: Recovery of recombinant Mycobacterium tuberculosis antigens fused with cell wall-anchoring motif (LysM) from inclusion bodies using non-denaturing reagent (N-laurylsarcosine)
Source: BMC Biotechnol. 2019 May 14;19:27. doi: 10.1186/s12896-019-0522-x (PMC6518676; doi:10.1186/s12896-019-0522-x)
Supplement: Supplementary file 1 — Figure S1. Confirmation of ARL and AR insert in pRSF:Duet plasmid by double restriction enzyme digests. (DOCX 692 kb) [file 12896_2019_522_MOESM1_ESM.docx]

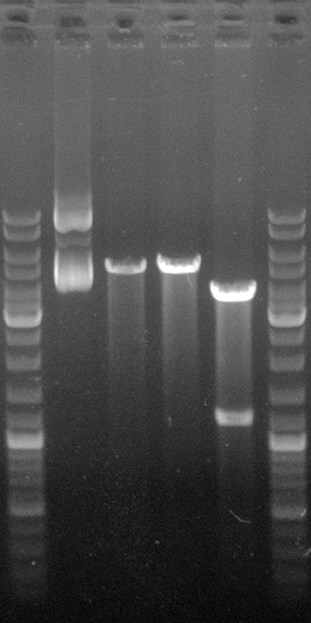


4

3

M

2

1

M

**(A)**

0.5 kb

1.0 kb

1.2 kb

~1.3 kb

2

1

M

**(B)**


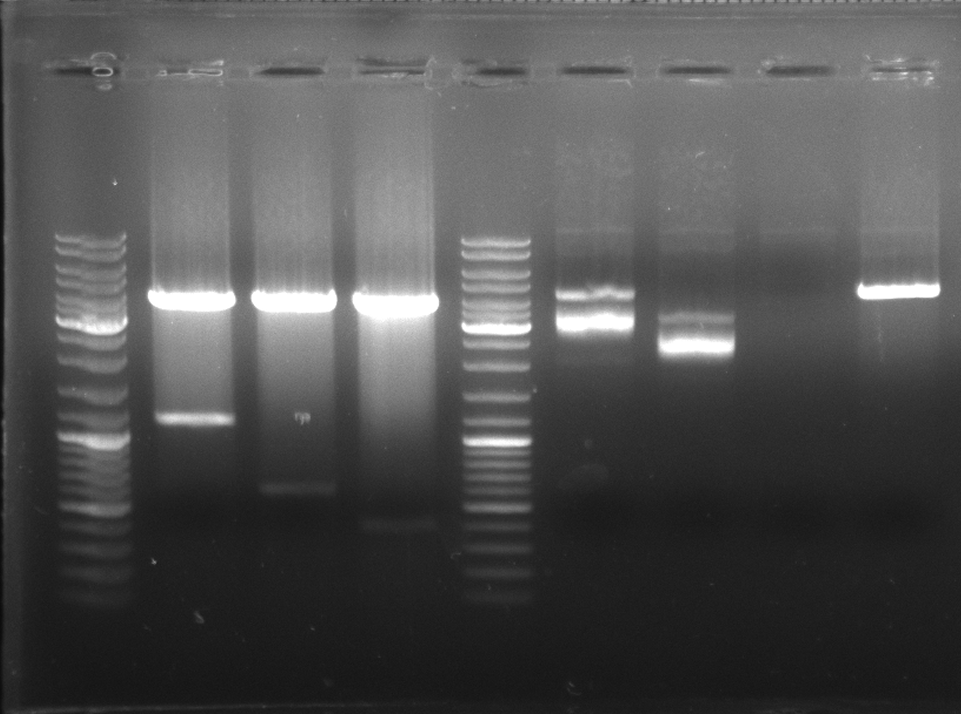


1.2 kb

0.5 kb

1.0 kb

~0.6 kb

**Figure S1:** (A) Digestion profile of plasmid extracted from transformants harbouring pRSF:ARL. Lane M: Ladder Mix (Fermentas, Canada). Lane 1: undigested plasmid pRSF:ARL, Lane 2: *Bam*HI single digestion; Lane 3: *Not*I single digestion; Lane 4: Double digestion with *Bam*HI/*Not*I. The double digestion for pRSF:ARL liberated gene of insert, ARL at expected size of 1289 bp. The restriction enzyme analysis confirmed *E. coli* Rossetta harbours the target pRSF:ARL plasmid. (B) Digestion profile of plasmid extracted from transformants harbouring pRSF:AR. Lane M: Ladder Mix (Fermentas, Canada). Lane 1: Double digestion with *Bam*HI/*Not*I of positive control that ensured gene liberated at 1200 bp; Lane 2: The double digestion with *Bam*HI/*Not*I of pRSF:AR liberated gene of insert, AR at expected size of 613bp. The restriction enzyme analysis confirmed *E. coli* Rossetta harbours the target pRSF:AR plasmids. Further verification of the extracted plasmid was performed with DNA sequence analysis for both pRSF:ARL and pRSF:AR.
