# Supplementary material for: Recovery of recombinant Mycobacterium tuberculosis antigens fused with cell wall-anchoring motif (LysM) from inclusion bodies using non-denaturing reagent (N-laurylsarcosine)
Source: BMC Biotechnol. 2019 May 14;19:27. doi: 10.1186/s12896-019-0522-x (PMC6518676; doi:10.1186/s12896-019-0522-x)
Supplement: Supplementary file 2 — Figure S2. Sequencing results for ARL and AR in pRSF:Duet plasmid. (DOCX 48 kb) [file 12896_2019_522_MOESM2_ESM.docx]

| **(A)** | | | | |
| --- | --- | --- | --- | --- |
| **Score** | **Expect** | **Identities** | **Gaps** | **Strand** |
| 2298 bits(1244) | 0.0 | 1244/1244(100%) | 0/1244(0%) | Plus/Plus |

Forward ARL

Query 1 ATGGGCAGCAGCCATCACCATCATCACCACAGCCA**GGATCCGAATTCG**CTGACCAGCGAG 60

||||||||||||||||||||||||||||||||||||||||||||||||||||||||||||

Sbjct 1 ATGGGCAGCAGCCATCACCATCATCACCACAGCCAGGATCCGAATTCGCTGACCAGCGAG 60

Query 61 CTGCCGCAATGGTTGTCCGCCAACAGGGCCGTGAAGCCCACCGGCAGCGCTGCAATCGGC 120

||||||||||||||||||||||||||||||||||||||||||||||||||||||||||||

Sbjct 61 CTGCCGCAATGGTTGTCCGCCAACAGGGCCGTGAAGCCCACCGGCAGCGCTGCAATCGGC 120

Query 121 TTGTCGATGGCCGGCTCGTCGGCAATGATCTTGGCCGCCTACCACCCCAGATCTGATATC 180

||||||||||||||||||||||||||||||||||||||||||||||||||||||||||||

Sbjct 121 TTGTCGATGGCCGGCTCGTCGGCAATGATCTTGGCCGCCTACCACCCCAGATCTGATATC 180

Query 181 **AAGCTT**ACGCACAGCTGGGAGTACTGGGGCGCTCAACTCAACGCCATGAAGAGATCTGAT 240

||||||||||||||||||||||||||||||||||||||||||||||||||||||||||||

Sbjct 181 AAGCTTACGCACAGCTGGGAGTACTGGGGCGCTCAACTCAACGCCATGAAGAGATCTGAT 240

Query 241 ATCGAGCTCAACCTGCGTGAGCGTGCGGAGGAGACTCGTACGGACACCCGCAGCCGGGTC 300

||||||||||||||||||||||||||||||||||||||||||||||||||||||||||||

Sbjct 241 ATCGAGCTCAACCTGCGTGAGCGTGCGGAGGAGACTCGTACGGACACCCGCAGCCGGGTC 300

Query 301 GAGGAGAGCCGTGCTCGCCTGACCAAGAGATCTGATATCCCATGGCGGCTGGAAGACGAG 360

||||||||||||||||||||||||||||||||||||||||||||||||||||||||||||

Sbjct 301 GAGGAGAGCCGTGCTCGCCTGACCAAGAGATCTGATATCCCATGGCGGCTGGAAGACGAG 360

Query 361 ATGAAAGAGGGGCGCTACGAGGTACGCGCGGAGCTTCCCGGGGTCGACCCCGACAAGGAC 420

||||||||||||||||||||||||||||||||||||||||||||||||||||||||||||

Sbjct 361 ATGAAAGAGGGGCGCTACGAGGTACGCGCGGAGCTTCCCGGGGTCGACCCCGACAAGGAC 420

Query 421 GTCGACATTATGGTCCGCGATGGTCAGCTGACCATCAAGGCCGAGCGCACCGAGCAGAAG 480

||||||||||||||||||||||||||||||||||||||||||||||||||||||||||||

Sbjct 421 GTCGACATTATGGTCCGCGATGGTCAGCTGACCATCAAGGCCGAGCGCACCGAGCAGAAG 480

Query 481 GACTTCGACGGTCGCTCGGAATTCGCGTACGGTTCCTTCGTTCGCACGGTGTCGCTGCCG 540

||||||||||||||||||||||||||||||||||||||||||||||||||||||||||||

Sbjct 481 GACTTCGACGGTCGCTCGGAATTCGCGTACGGTTCCTTCGTTCGCACGGTGTCGCTGCCG 540

Query 541 GTAGGTGCTGACGAGGACGACATTAAGGCCACCTACGACAAGGGCATTCTTACTGTGTCG 600

||||||||||||||||||||||||||||||||||||||||||||||||||||||||||||

Sbjct 541 GTAGGTGCTGACGAGGACGACATTAAGGCCACCTACGACAAGGGCATTCTTACTGTGTCG 600

Forward *lys*M

Query 601 GTGGCGGTTTCGGAAGGGAAGCCA**AGATCTGATATC**CTGCAGGGTACCACTACTTATACC 660

||||||||||||||||||||||||||||||||||||||||||||||||||||||||||||

Sbjct 601 GTGGCGGTTTCGGAAGGGAAGCCAAGATCTGATATCCTGCAGGGTACCACTACTTATACC 660

Reverse AR

Query 661 GTCAAATCTGGTGATACTCTTTGGGGAATCTCACAAAGATATGGAATTAGTGTCGCTCAA 720

||||||||||||||||||||||||||||||||||||||||||||||||||||||||||||

Sbjct 661 GTCAAATCTGGTGATACTCTTTGGGGAATCTCACAAAGATATGGAATTAGTGTCGCTCAA 720

Query 721 ATTCAAAGTGCGAATAATCTTAAAAGTACCATTATCTACATTGGTCAAAAACTTGTACTG 780

||||||||||||||||||||||||||||||||||||||||||||||||||||||||||||

Sbjct 721 ATTCAAAGTGCGAATAATCTTAAAAGTACCATTATCTACATTGGTCAAAAACTTGTACTG 780

Query 781 ACAGGTTCAGCTTCTTCTACAAATTCAGGTGGTTCAAACAATTCCGCAAGCACTACTCCA 840

||||||||||||||||||||||||||||||||||||||||||||||||||||||||||||

Sbjct 781 ACAGGTTCAGCTTCTTCTACAAATTCAGGTGGTTCAAACAATTCCGCAAGCACTACTCCA 840

Query 841 ACCACTTCTGTGACACCTGCAAAACCAACTTCACAAACAACTGTTAAGGTTAAATCCGGA 900

||||||||||||||||||||||||||||||||||||||||||||||||||||||||||||

Sbjct 841 ACCACTTCTGTGACACCTGCAAAACCAACTTCACAAACAACTGTTAAGGTTAAATCCGGA 900

Query 901 GATACCCTTTGGGCGCTATCAGTAAAATATAAAACTAGTATTGCTCAATTGAAAAGTTGG 960

||||||||||||||||||||||||||||||||||||||||||||||||||||||||||||

Sbjct 901 GATACCCTTTGGGCGCTATCAGTAAAATATAAAACTAGTATTGCTCAATTGAAAAGTTGG 960

Query 961 AATCATTTAAGTTCAGATACCATTTATATTGGTCAAAATCTTATTGTTTCACAATCTGCT 1020

||||||||||||||||||||||||||||||||||||||||||||||||||||||||||||

Sbjct 961 AATCATTTAAGTTCAGATACCATTTATATTGGTCAAAATCTTATTGTTTCACAATCTGCT 1020

Query 1021 GCTGCTTCAAATCCTTCGACAGGTTCAGGCTCAACTGCTACCAATAACTCAAACTCGACT 1080

||||||||||||||||||||||||||||||||||||||||||||||||||||||||||||

Sbjct 1021 GCTGCTTCAAATCCTTCGACAGGTTCAGGCTCAACTGCTACCAATAACTCAAACTCGACT 1080

Query 1081 TCTTCTAACTCAAATGCCTCAATTCATAAGGTCGTTAAAGGAGATACTCTCTGGGGACTT 1140

||||||||||||||||||||||||||||||||||||||||||||||||||||||||||||

Sbjct 1081 TCTTCTAACTCAAATGCCTCAATTCATAAGGTCGTTAAAGGAGATACTCTCTGGGGACTT 1140

Query 1141 TCGCAAAAATCTGGCAGCCCAATTGCTTCAATCAAGGCTTGGAATCATTTATCTAGCGAT 1200

||||||||||||||||||||||||||||||||||||||||||||||||||||||||||||

Sbjct 1141 TCGCAAAAATCTGGCAGCCCAATTGCTTCAATCAAGGCTTGGAATCATTTATCTAGCGAT 1200

Query 1201 ACTATTTTAATTGGTCAGTATCTACGAATAAAATAAGCGGCCGC 1244

||||||||||||||||||||||||||||||||||||||||||||

Sbjct 1201 ACTATTTTAATTGGTCAGTATCTACGAATAAAATAAGCGGCCGC 1244

Reverse *lys*M

**(B)**

| **Score** | **Expect** | **Identities** | **Gaps** | **Strand** |
| --- | --- | --- | --- | --- |
| 1197 bits(648) | 0.0 | 648/648(100%) | 0/648(0%) | Plus/Plus |

Forward ARL

Query 1 ATGGGCAGCAGCCATCACCATCATCACCACAGCCAGGATCCGAATTCGCTGACCAGCGAG 60

||||||||||||||||||||||||||||||||||||||||||||||||||||||||||||

Sbjct 1 ATGGGCAGCAGCCATCACCATCATCACCACAGCCAGGATCCGAATTCGCTGACCAGCGAG 60

Query 61 CTGCCGCAATGGTTGTCCGCCAACAGGGCCGTGAAGCCCACCGGCAGCGCTGCAATCGGC 120

||||||||||||||||||||||||||||||||||||||||||||||||||||||||||||

Sbjct 61 CTGCCGCAATGGTTGTCCGCCAACAGGGCCGTGAAGCCCACCGGCAGCGCTGCAATCGGC 120

Query 121 TTGTCGATGGCCGGCTCGTCGGCAATGATCTTGGCCGCCTACCACCCCAGATCTGATATC 180

||||||||||||||||||||||||||||||||||||||||||||||||||||||||||||

Sbjct 121 TTGTCGATGGCCGGCTCGTCGGCAATGATCTTGGCCGCCTACCACCCCAGATCTGATATC 180

Query 181 **AAGCTT**ACGCACAGCTGGGAGTACTGGGGCGCTCAACTCAACGCCATGAAGAGATCTGAT 240

||||||||||||||||||||||||||||||||||||||||||||||||||||||||||||

Sbjct 181 AAGCTTACGCACAGCTGGGAGTACTGGGGCGCTCAACTCAACGCCATGAAGAGATCTGAT 240

Query 241 ATCGAGCTCAACCTGCGTGAGCGTGCGGAGGAGACTCGTACGGACACCCGCAGCCGGGTC 300

||||||||||||||||||||||||||||||||||||||||||||||||||||||||||||

Sbjct 241 ATCGAGCTCAACCTGCGTGAGCGTGCGGAGGAGACTCGTACGGACACCCGCAGCCGGGTC 300

Query 301 GAGGAGAGCCGTGCTCGCCTGACCAAGAGATCTGATATCCCATGGCGGCTGGAAGACGAG 360

||||||||||||||||||||||||||||||||||||||||||||||||||||||||||||

Sbjct 301 GAGGAGAGCCGTGCTCGCCTGACCAAGAGATCTGATATCCCATGGCGGCTGGAAGACGAG 360

Query 361 ATGAAAGAGGGGCGCTACGAGGTACGCGCGGAGCTTCCCGGGGTCGACCCCGACAAGGAC 420

||||||||||||||||||||||||||||||||||||||||||||||||||||||||||||

Sbjct 361 ATGAAAGAGGGGCGCTACGAGGTACGCGCGGAGCTTCCCGGGGTCGACCCCGACAAGGAC 420

Query 421 GTCGACATTATGGTCCGCGATGGTCAGCTGACCATCAAGGCCGAGCGCACCGAGCAGAAG 480

||||||||||||||||||||||||||||||||||||||||||||||||||||||||||||

Sbjct 421 GTCGACATTATGGTCCGCGATGGTCAGCTGACCATCAAGGCCGAGCGCACCGAGCAGAAG 480

Query 481 GACTTCGACGGTCGCTCGGAATTCGCGTACGGTTCCTTCGTTCGCACGGTGTCGCTGCCG 540

||||||||||||||||||||||||||||||||||||||||||||||||||||||||||||

Sbjct 481 GACTTCGACGGTCGCTCGGAATTCGCGTACGGTTCCTTCGTTCGCACGGTGTCGCTGCCG 540

Query 541 GTAGGTGCTGACGAGGACGACATTAAGGCCACCTACGACAAGGGCATTCTTACTGTGTCG 600

||||||||||||||||||||||||||||||||||||||||||||||||||||||||||||

Sbjct 541 GTAGGTGCTGACGAGGACGACATTAAGGCCACCTACGACAAGGGCATTCTTACTGTGTCG 600

Query 601 GTGGCGGTTTCGGAAGGGAAGCCAAGATCTGATATCCTGCAGGGTACC 648

||||||||||||||||||||||||||||||||||||||||||||||||

Sbjct 601 GTGGCGGTTTCGGAAGGGAAGCCAAGATCTGATATCCTGCAGGGTACC 648

Reverse AR

**Figure S2:** (A) Alignment statistics for match between Query (pRSF:ARL construct) and Sbjct (pRSF:ARL of template reference). (B) Alignment statistics for match between Query (pRSF:AR construct) and Sbjct (pRSF:AR of template reference). Both alignments showed 100% similarity between the constructed insert of ARL and AR in pRSF:Duet-1 with the template reference sequences, respectively. DNA sequences highlighted in represent the Ag85B_101-115,126-140,261-275_ epitopic sequences, linker sequence, restriction enzymes sequence, Rv0475_34-59_ and Rv2031_41-70,95-108_ epitopic sequences and xxx *lys*M binding domain sequence. The arrows represent the forward or reverse primers corresponding to ARL, AR or *lys*M DNA sequences.
