# Supplementary material for: Recovery of recombinant Mycobacterium tuberculosis antigens fused with cell wall-anchoring motif (LysM) from inclusion bodies using non-denaturing reagent (N-laurylsarcosine)
Source: BMC Biotechnol. 2019 May 14;19:27. doi: 10.1186/s12896-019-0522-x (PMC6518676; doi:10.1186/s12896-019-0522-x)
Supplement: Supplementary file 3 — Figure S3. Western blot analysis of ARL from the total protein fraction sample. (DOCX 60 kb) [file 12896_2019_522_MOESM3_ESM.docx]

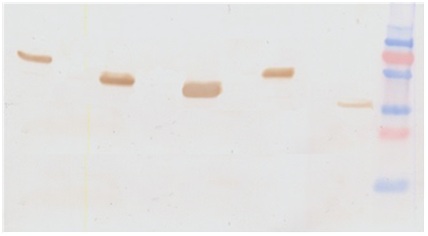


15

25

35

55

75

**MW (kDa)**

M

(1)

(2)

(3)

ARL

**Figure S3**: Western blot analysis of total lysed protein of ARL post IPTG induction using anti-his monoclonal primary antibody (1:1000) (0.2 µg/µl) (Novagen, USA) and goat anti-mouse IgG-HRP (Calbiochem, USA) as the secondary antibody. The recombinant ARL fusion protein showed expected protein band sizes of 45 kDa as indicated by the arrow. Lane M: PageRuler™ Prestained Plus Protein Ladder (Fermentas, Canada); Lane 1: positive control of purified protein of 55 kDa; Lanes 2: positive control of purified protein of 37 kDa; Lanes 3: total lysed fraction of ARL post IPTG induction.
